# Association Between Social Adaptability Index Score and Lifetime Criminal Legal Involvement in U.S. Adults

**DOI:** 10.1089/heq.2021.0110

**Published:** 2022-03-15

**Authors:** Laura C. Hawks, Rebekah J. Walker, Leonard E. Egede

**Affiliations:** ^1^ Division of General Internal Medicine, Medical College of Wisconsin, Milwaukee, Wisconsin, USA.; ^2^ Center for Advancing Population Science, Medical College of Wisconsin, Milwaukee, Wisconsin, USA.

**Keywords:** criminal justice involvement, health services research, incarceration, social determinants of health, social risk

## Abstract

**Background:**

Exposure to the criminal legal system is associated with negative health outcomes and profound socioeconomic health disparities. The social adaptability index (SAI) is a validated composite scale based on five indicators of socioeconomic status; a higher score predicts better health outcomes. However, little is known about the relationship between cumulative social risk factors as measured by the SAI and lifetime criminal legal involvement (CLI).

**Methods:**

Using a cross-sectional, nationally representative sample of U.S. adults, we calculated SAI score by lifetime CLI status, and used logistic regression with predictive margins to calculate risk of lifetime CLI by SAI quartile adjusting for demographic and clinical covariates.

**Results:**

A total of 213,678 participants were included, among whom 16.8% reported lifetime CLI. Mean SAI score was lower among those with lifetime CLI compared with those without (7.77, 95% confidence interval [CI]: 7.72–7.83 vs. 8.52, 95% CI: 8.50–8.55). There was a linear association between SAI quartile and predicted probability of lifetime CLI: first quartile: 23.9% (95% CI: 23.0–24.7); second quartile: 19.2% (95% CI: 18.6–19.8); third quartile: 17.5% (95% CI: 16.9–18.1); and fourth quartile: 12.5% (95% CI: 12.1–13.0).

**Conclusion:**

The SAI score is associated in a reverse linear manner with lifetime risk of CLI, suggesting that to successfully improve health outcomes among those with CLI, interventions may need to target multiple SAI components simultaneously. Interventions that successfully position individuals to achieve higher social adaptability by targeting multiple factors may reduce the health-harming effects of exposure to the criminal legal system.

## Introduction

The United States has the largest correctional control system in the world.^1^ In addition to the 2 million adults incarcerated at any given time, 4.4 million adults are surveilled under community supervision,^2^ and 10.5 million are arrested every year.^3^ The health impacts of exposure to the criminal legal system are far-reaching. Exposure to incarceration and community supervision is associated with increased risk of mortality,^4,5^ while even a single police stop is associated with worse overall and mental health.^6,7^ Risk of criminal legal involvement (CLI) and its health consequences are associated with drastic racial and socioeconomic health disparities.^8^ However, little is known about how the accumulation of social risk factors may contribute to the health-harming effects of CLI and the health disparities to which it contributes.

The social adaptability index (SAI) is a composite indicator validated to predict risk for health outcomes based on socioeconomic status and was designed to overcome the limitations of cruder but more commonly used correlates, including race, gender, or geographic location.^9^ The components of the SAI include employment, education, marital status, substance use, and income; a higher SAI score indicates higher socioeconomic status.^9^ A low SAI score has been shown to predict poor health outcomes (such as graft failure in kidney transplant^10^) and mortality in the overall population and in subpopulations of patients with chronic disease, including diabetes.^9,11^ Most elements of the SAI are modifiable, suggesting potential areas of intervention further upstream in a patient's life course, which may ultimately improve health outcomes.

Prior work has shown that CLI is associated with the five individual components of the SAI^12^; for example, low income and lack of education are predisposing factors for CLI, as are high rates of substance use.^8^ Moreover, studies show that those from disadvantaged backgrounds experience a greater degree of socioeconomic decline after exposure to the criminal legal system, creating compounding layers of social risk.^13^ However, the use of a composite indicator among those with CLI, such as SAI, which could be easily captured with limited data, has not been investigated.

Understanding the relationship between lifetime CLI and the SAI may help us identify potential points of intervention that may lead to improved health outcomes among those with CLI. Therefore, we used cross-sectional nationally representative data to test the association between lifetime CLI and SAI score, in sum, by quartile, and by component. We hypothesized that a higher total SAI score, and higher score by each individual component of the SAI, would be associated with reduced likelihood of lifetime CLI.

## Methods

To conduct this study, we used publicly available survey data from the National Survey of Drug Use and Health (NSDUH) including years 2015–2019. The NSDUH data contain nationally representative cross-sectional data and are conducted each year by the Substance Abuse and Mental Health Services Administration to provide estimates for rates of substance use and mental health conditions among adolescents and adults in the United States. In addition, the NSDUH contains questions regarding some chronic diseases, socioeconomic factors, and past CLI.

The design of this survey excludes persons in institutional settings, meaning that those who are currently incarcerated or living in long-term care facilities cannot be interviewed. The survey is conducted by an in-person interview; however, respondents provide most responses anonymously into a computer.^14^

### Population

Our sample included all adults aged 18 or older who provided a response to a question regarding lifetime exposure to the criminal legal system (*N*=213,678).

### Main measures

Our exposure variable was lifetime involvement in the criminal legal system. We considered individuals to have lifetime CLI if they responded “yes” to the question, “Not counting minor traffic violations, have you ever been arrested and booked for breaking the law?” Missing data were excluded from the analysis.

#### Outcome

We created a composite ordinal variable capturing a respondent's SAI score using questions provided in the survey that matched the validated scale.^9^ The variables and scoring matched the previously validated SAI, with the exceptions noted below. All variations from the validated SAI are due to lack of available data.

- Employment: 0 points—unemployed; 1 point—retired; 2 points—part-time employed; 3 points—full-time employed.
- Education: 0 points—no high school graduation; 1 point—high school graduation; 2 points—college graduate (validated SAI includes 3 points for postcollege education or doctorate degree, not included in this study).
- Marital status: 0—never married; 1—widowed/divorced; 2—married (validated SAI includes 3 points for married with children, not included in this study).
- Substance use: 0 points—tobacco, alcohol, and drug use disorder; 1 point—use disorder of two of three substances; 2 points—use disorder of three substances; 3—no tobacco, alcohol, or drug use disorder.
- Income: 0 points—<$20,000 annual household income; 1 point—$20,000–50,000 annual household income; 2 points—>$50,000 annual household income.

The SAI was calculated for each respondent by adding all five components, for a final scale with a range of 0–12. Lower scores indicated a higher social risk. Following creation of the continuous scale, we broke the SAI into quartiles, and included each of the five components of the SAI as a separate outcome in addition to the overall scale. Therefore, six outcomes were investigated: (1) SAI quartile, (2) employment, (3) education, (4) marital status, (5) substance use, and (6) income.

#### Covariates

To account for a range of sociodemographic and clinical confounders, our models included several covariates. Demographic covariates included the following: age category (18–25, 26–34, 35–49, 50+); sex (male, female); race/ethnicity (white, non-Hispanic; black, non-Hispanic; Hispanic, other, including non-Hispanic Native American/Alaskan native, non-Hispanic native Hawaiian or Pacific Islander, non-Hispanic Asian, or more than one race); and insurance coverage (Medicare, Medicaid, VA health care, private, none).

We included all self-reported physical medical conditions addressed in the survey as independent dichotomous variables, including diabetes, high blood pressure, heart condition, kidney disease, asthma, chronic obstructive pulmonary disease, cirrhosis, hepatitis B/C, HIV/AIDS, and cancer. We selected covariates based on prior literature to ensure that we accounted for factors that may influence the relationship between CLI and SAI components, to establish evidence supporting an independent association between the two.

### Statistical analyses

We first calculated descriptive statistics to report the sociodemographic characteristics and health conditions of the entire sample and by prior CLI exposure. Second, we calculated the unadjusted mean SAI score and 95% confidence interval (CI) by prior CLI exposure. We then calculated the unadjusted score for each SAI indicator (employment, education, marital status, substance use, and income) along with 95% CI. Third, we estimated the adjusted predicted probability of prevalence of lifetime CLI by SAI quartile. To quantify the adjusted odds of lifetime CLI and SAI quartile, we used multivariable logistic regression models that controlled for age, sex, race/ethnicity, health insurance coverage, and clinical comorbidities.

We then reported predicted marginal effects (and 95% CIs) at representative values and pairwise comparisons of the adjusted odds ratios (aORs) of CLI comparing each quartile from multivariate logistic regression models.

Finally, using multivariate logistic regression controlling for age, sex, race/ethnicity, health insurance coverage, and clinical comorbidities, we calculated the adjusted odds of experiencing lifetime CLI by each level of SAI indicator: (1) employment (unemployed, retired, part-time employed, full-time employed); (2) education (less than high school, high school graduate, college graduate); (3) marital status (never married, widow/divorced, married); (4) substance use (tobacco, alcohol, and drug use disorder; use disorder for two of three substances; use disorder for one of three substances; no tobacco, alcohol, or drug use disorder); or (5) income (<$20,000, $20–50,000, >$50,000).

We used survey commands in Stata version 16 (Stata Corp LP, College Station, TX) to perform all analyses. The Substance Abuse and Mental Health Services Administration provides survey weights that account for complex survey design and allow extrapolation for the U.S. population, which we used for all analytic procedures.^14^ We used two-sided *p*-values of <0.05 to determine statistical significance.

## Results

A total of 213,678 participants were included in the unweighted sample, representing 270,582,507 U.S. adults. Among respondents, 16.8% reported lifetime CLI. There were substantial demographic differences between those with and without lifetime CLI. Those with lifetime CLI were more likely to be in the middle age categories (26–35 or 35–49) compared with those with never CLI. Respondents who identified as black were more likely to report lifetime CLI than any other race group (15.1% vs. white, 11.1%). Respondents reported substantial differences in most measures of socioeconomic status. Full data on demographic and comorbid conditions are included in Table 1.

**Table 1.** Demographics, Components of Social Adaptability Index, and Comorbidities by Prior Criminal Legal Involvement Among U.S. Adults, 2015–2019

	Total population (N=213,678), %	Criminal legal involvement (N=37,279), %	No criminal legal involvement (N=176,399), %	p
Population	100	16.8	83.1	
Demographics				
Age, years				<0.001
18–25	13.9	9.5	14.8	
26–34	15.9	19.7	15.2	
35–49	24.6	30.6	23.4	
50+	45.4	40.0	46.5	
Sex				<0.001
Male	48.2	70.9	43.5	
Female	51.8	29.0	56.4	
Race				<0.001
White, non-Hispanic	63.9	65.3	63.6	
Black, non-Hispanic	11.2	15.1	11.1	
Hispanic	16.4	14.0	16.4	
Other	8.7	5.4	8.7	
Employment				<0.001
Full-time employed	49.4	54.3	48.4	
Part-time employed	13.0	10.2	13.6	
Unemployed	4.3	6.7	3.8	
Other	33.1	29.7	34.0	
Education				<0.001
Less than high school	12.6	16.4	11.8	
High school diploma	24.8	30.0	23.7	
Some college	30.8	34.5	30.1	
College degree or beyond	31.6	18.9	34.2	
Marital status				<0.001
Never married	51.5	40.9	53.6	
Widowed, divorced, separated	19.6	25.0	18.5	
Married	28.8	34.0	27.8	
Annual household income				<0.001
<100% federal poverty level	14.0	18.9	13.0	
100–200% federal poverty level	19.7	22.9	19.1	
>200% federal poverty level	66.2	58.1	67.8	
Health insurance				
Private	66.6	53.9	69.1	<0.001
Medicare	22.4	17.0	23.5	<0.001
Medicaid	14.4	21.7	13.0	<0.001
VA health	5.1	6.1	4.9	<0.001
Uninsured	9.9	15.3	8.8	<0.001
Comorbidities				
Diabetes	10.6	10.7	10.6	0.8824
Heart condition	10.5	10.9	10.4	0.04
Hypertension	19.6	18.6	19.8	0.001
Kidney disease	1.9	1.7	2.0	0.07
COPD	4.3	6.5	3.9	<0.001
Asthma	9.4	9.7	9.4	0.20
Cirrhosis	0.3	0.6	0.2	<0.001
Hepatitis B/C	1.3	3.3	0.8	<0.001
HIV/AIDS	0.2	0.5	0.1	<0.001
Cancer	6.3	5.0	6.5	<0.001
Past year any mental illness	19.0	26.4	17.5	<0.001

COPD, chronic obstructive pulmonary disease; VA, Veteran's Affairs.

In Table 2, we present the mean SAI score by lifetime CLI status. Those with lifetime CLI had a lower SAI score compared with those without lifetime CLI (7.77, 95% CI: 7.72–7.83 vs. 8.52, 95% CI: 8.50–8.55), indicating lower socioeconomic status. Furthermore, those with lifetime CLI reported a lower score for most SAI indicators (education: 1.02 vs. 1.22, *p*<0.001; marital status: 1.06 vs. 1.25; *p*<0.001; substance use *p*=2.60 vs. 2.84; *p*<0.001; and income 1.24 vs. 1.40; *p*<0.001), except for employment (1.79 vs. 1.76, *p*=0.02).

**Table 2.** Mean Social Adaptability Index Score, Total and for Each Component, by Prior Criminal Legal Involvement, U.S. Adults, 2015–2019

	Criminal legal involvement	No criminal legal involvement	p
	Mean score (95% CI)	Mean score (95% CI)	
Social adaptability index	7.77 (7.72–7.83)	8.52 (8.50–8.55)	<0.001
Employment	1.79 (1.77–1.81)	1.76 (1.75–1.77)	0.02
Education (mean score)	1.02 (1.01–1.03)	1.22 (1.22–1.23)	<0.001
Marital status (mean score)	1.06 (1.05–1.08)	1.25 (1.24–1.26)	<0.001
Substance use (mean score)	2.60 (2.59–2.61)	2.84 (2.84–2.84)	<0.001
Income (mean score)	1.24 (1.22–1.25)	1.40 (1.40–1.41)	<0.001

CI, confidence interval.

In Table 3, we present the predicated probability of prevalence of CLI by SAI quartile. The prevalence of CLI decreases with each SAI quartile increase. After adjustment for age, sex, race/ethnicity, health insurance coverage, and comorbidities, the prevalence of lifetime CLI was 23.9% (95% CI: 23.0–24.7%) in the first quartile, 19.2% (95% CI: 18.6–19.8%) in the second quartile, 17.5% (95% CI: 16.9–18.1%) in the third quartile, and 12.5% (95% CI: 12.1–13.0%) in the fourth quartile. The adjusted odds of lifetime CLI for those in the first SAI quartile versus those in the fourth SAI quartile were 2.38 (95% CI: 2.21–2.25).

**Table 3.** Predicted Probability of Prevalence of Lifetime Criminal Legal Involvement by Social Adaptability Index Quartile

	Predicted prevalence of CLI, % (95% CI)	Lifetime CLI (aOR) vs. quartile 1^**^	Lifetime CLI (aOR) vs. quartile 2^**^	Lifetime CLI (aOR) vs. quartile 3^**^	Lifetime CLI (aOR) vs. quartile 4^**^
SAI quartile					
1 (≤5)	23.9 (23.0–24.7)	REF	1.36 (1.29–1.44)	1.55 (1.45–1.65)	2.38 (2.21–2.55)
2 (6–7)	19.2 (18.6–19.8)	0.73 (0.69–0.77)	REF	1.13 (1.06–1.20)	1.74 (1.63–1.84)
3 (8–9)	17.5 (16.9–18.1)	0.64 (0.60–0.68)	0.88 (0.83–0.93)	REF	1.53 (1.44–1.63)
4 (≥10)	12.5 (12.1–13.0)	0.42 (0.39–0.45)	0.57 (0.54–0.60)	0.65 (0.61–0.69)	REF

Pairwise comparisons of lifetime CLI aORs.

^*^ All models adjusted for age, sex, race/ethnicity, health insurance coverage, and comorbidities among U.S. adults.

^**^ All pairwise comparisons across SAI quartiles were significant at *p*<0.001.

aOR, adjusted odds ratio; CLI, criminal legal involvement; and SAI, social adaptability index.

**FIG. 1.**
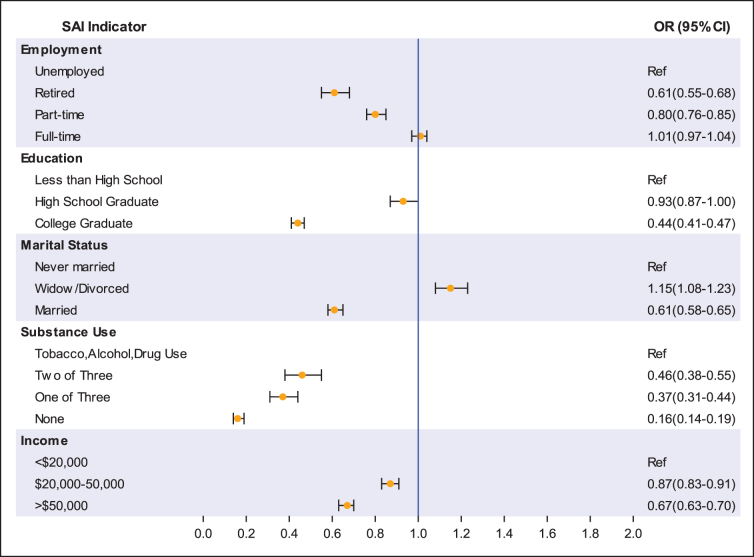
Adjusted OR and 95% CIs for lifetime criminal legal involvement by SAI component. CI, confidence interval; OR, odds ratio.

In Figure 1, we present the odds ratio adjusted for age, sex, race/ethnicity, health insurance coverage, and comorbidities for each SAI component. With the lowest score for each indicator serving as the referent group, all components functioned as protective factors for lifetime CLI, except for being widow/divorced, which was a risk factor (referent never married; aOR: 1.15; 95% CI: 1.08–1.23), and having full-time employment, which had no association (referent unemployed; aOR 1.01; 95% CI: 0.97–1.04).

### Health equity implications

In this nationally representative sample, we found that one in six adults report lifetime exposure to the criminal legal system, and that the previously validated SAI score was lower for adults with prior CLI, predicting poor health outcomes. The results show that as the SAI score increases, the likelihood of lifetime CLI decreases. Furthermore, when investigated by an individual component, each was associated with a higher likelihood of CLI, with the exception of current employment. Overall, these findings underscore the need to consider multiple risk factors when considering interventions to improve outcomes for those with CLI, posited to be an important driver of racial and socioeconomic health disparities.^8^

While there is robust evidence to support the association between socioeconomic status and risk for CLI, our findings indicate the cumulative effect of layering social risks on CLI, with those in the first (lowest) SAI quartile experiencing more than twice the odds of lifetime CLI compared with those in the fourth. Importantly, given the linear relationship between the overall SAI score and lifetime CLI risk, our findings suggest the need to address these protective factors using a multipronged approach targeting multiple components of social adaptability simultaneously rather than examining an individual component, which has been the focus of the majority of work to this point.

Two interventions specifically designed to improve the health of those with recent CLI that have targeted multiple SAI indicators and can be built on further are the Transitions Clinic Network and the Culture of Health. The Transitions Clinic Network employs a community health worker with prior legal involvement embedded within a primary care clinic to help patients navigate the health care system, support chronic disease management, and facilitate access to basic needs services.^15^ As such, this model targets multiple SAI indicators (treatment for addiction, income, employment, and education). While specific disease outcomes have not been studied, it has been shown to reduce emergency room visits and some measures of future legal involvement (days incarcerated, number of technical violations of probation and parole).^16,17^

The second approach is called “Culture of Health,” a multidisciplinary intervention based out of community correction offices, which includes health care providers and social services. So far, only a pilot study has been completed; it noted “buy-in from probation officers and dwindling support from change team members” as barriers to implementation.^18,19^ Future research should consider approaches such as these that aim to broadly enhance social adaptability.

In addition to the multipronged interventions, our findings suggest specific areas of socioeconomic status that could serve as testable intervention points to ameliorate the health-harming effects of exposure to the criminal legal system, including obtaining a postsecondary degree, preventing and treating substance use disorders, and promoting access to a stable income. For example, our study found a direct linear association between substance use disorders and CLI, suggesting that ongoing tobacco, alcohol, and drug use may contribute to poor health outcomes in this population. Prior work has found that drug overdose, liver disease, and lung disease (all associated with increased substance use) are leading causes of morbidity and mortality among those currently or previously incarcerated.^4,20,21^

These findings underscore the importance of broadly implemented evidenced-based programs for the treatment and prevention of substance use disorders. In the United States, addiction is often untreated or undertreated. A study from 2015 found that only 10.8% of those with a substance use disorder received any treatment.^22^ For those involved in the criminal legal system, access to evidence-based treatment is especially poor.

For example, among those on probation with an active substance use disorder, nearly one-third did not receive treatment.^23^ For those who did receive treatment, it is unlikely that they received standard-of-care pharmacotherapy, which is known to both improve health outcomes and reduce future CLI.^24^ Broader implementation of evidence-based treatment for substance use disorder is needed as well as rigorous standards to mandate provision of standard-of-care treatment for correctional providers.

The directionality of the association between SAI components and lifetime CLI cannot be inferred from our analyses. However, a robust body of literature suggests that the relationship between CLI and SAI components is bidirectional. For example, while low educational attainment likely predisposes an individual to legal involvement, legal involvement in turn reduces the prospects for educational opportunities. Education has long been at the forefront for researchers interested in both health and criminal legal outcomes,^25^ and our study found a strong reverse association between receiving a college degree and lifetime CLI.

Studies have similarly shown that postsecondary education for incarcerated students reduces risk of recidvism.^26,27^ However, policy has made it hard for those with CLI to access higher education. For decades, federal law prohibited incarcerated students from accessing federal Pell grants to pursue a college degree, resulting in a decline of in-prison college programs from 772 to 8.^28^ Given this evidence supporting a bidirectional relationship, it is plausible that improving access to high-quality education for populations at high risk of CLI may both reduce future CLI and improve health outcomes, although further research is needed to substantiate this hypothesis.

While interventions that broadly improve the socioeconomic status for low-income Americans are necessary to reduce racial disparities within both our health care and criminal legal systems, they will likely prove insufficient in the absence of legal reform measures. Prior work has shown that communities of color are excessively policed^29^—disparities in punishment begin as early as elementary school.^30^

For well over a century—documented by sociologist W.E.B. Du Bois in the 1890s—black Americans have been arrested and incarcerated at rates far outpacing their proportion of the general population.^29,31^ Racial disparities in length of sentencing are also well documented.^32^ In short, structural racism in the design and operation of the criminal legal system itself contributes to the racial disparity in frequency and intensity of exposure to lifetime CLI and must be dismantled if we are to achieve equity in health and legal outcomes.

Our study has several limitations. The cross-sectional design of this study precludes causal association. In addition to the possibility of reverse causation addressed above, unmeasured confounders are possible. Nonetheless, it is worth considering that these may be understudied areas of potential intervention to prevent future CLI and improve health outcomes. We support further studies that can further elucidate causality and directionality of the associations presented here.

Second, our exposure variable is binary; due to the survey design, we are unable to capture a cumulative or qualitative effect of CLI. Prior work has suggested that cumulative time spent incarcerated has an increasingly deleterious effect on risk of death^33,34^; future research could consider the relationship between cumulative CLI and SAI. Third, the survey excludes individuals who are currently incarcerated, meaning that a substantial proportion of U.S. adults with CLI are not included in our exposed population. However, this limitation likely renders the findings more conservative.

## Conclusion

This study shows that the SAI score, which has been shown predict poor health outcomes, has direct linear association with lifetime CLI, which is also known to be associated with poor health outcomes. Further analyses suggest possible protective factors that may reduce the health-harming effects of lifetime CLI: college education, annual household income >$20,000, and prevention or treatment of substance use disorders. However, transformative interventions will likely need to target multiple SAI components simultaneously. Interventions that successfully position individuals to achieve higher social adaptability may improve health outcomes for the millions of Americans with prior involvement in our criminal legal system.

## Availability of the Supporting Data

The data sets supporting the conclusions of this article are publicly available from the NSDUH website: https://www.datafiles.samhsa.gov/study-series/national-survey-drug-use-and-health-nsduh-nid13517.

## Abbreviations Used

aOR: adjusted odds ratio
CI: confidence interval
CLI: criminal legal involvement
NSDUH: National Survey of Drug Use and Health
OR: odds ratio
SAI: social adaptability index
